# Supplementary material for: Neuronal differentiation and cell-cycle programs mediate response to BET-bromodomain inhibition in MYC-driven medulloblastoma
Source: Nat Commun. 2019 Jun 3;10:2400. doi: 10.1038/s41467-019-10307-9 (PMC6546744; doi:10.1038/s41467-019-10307-9)
Supplement: Supplementary file 1 — Supplementary Information [file 41467_2019_10307_MOESM1_ESM.pdf]

Neuronal differentiation and cell-cycle programs mediate response to BET-bromodomain inhibition in MYC-driven medulloblastoma

Bandopadhayay et al.

Supplementary Figure 1

A. Gene expression heat map of top 50 differentially expressed genes between D458 cells treated with DMSO or JQ1 at a concentration of 1μM for 24 hours.

B. Gene expression heat map of top 50 differentially expressed genes between D283 cells treated with DMSO or JQ1 at a concentration of 1μM for 24 hours.

Source data provided in Supplementary Table 1.

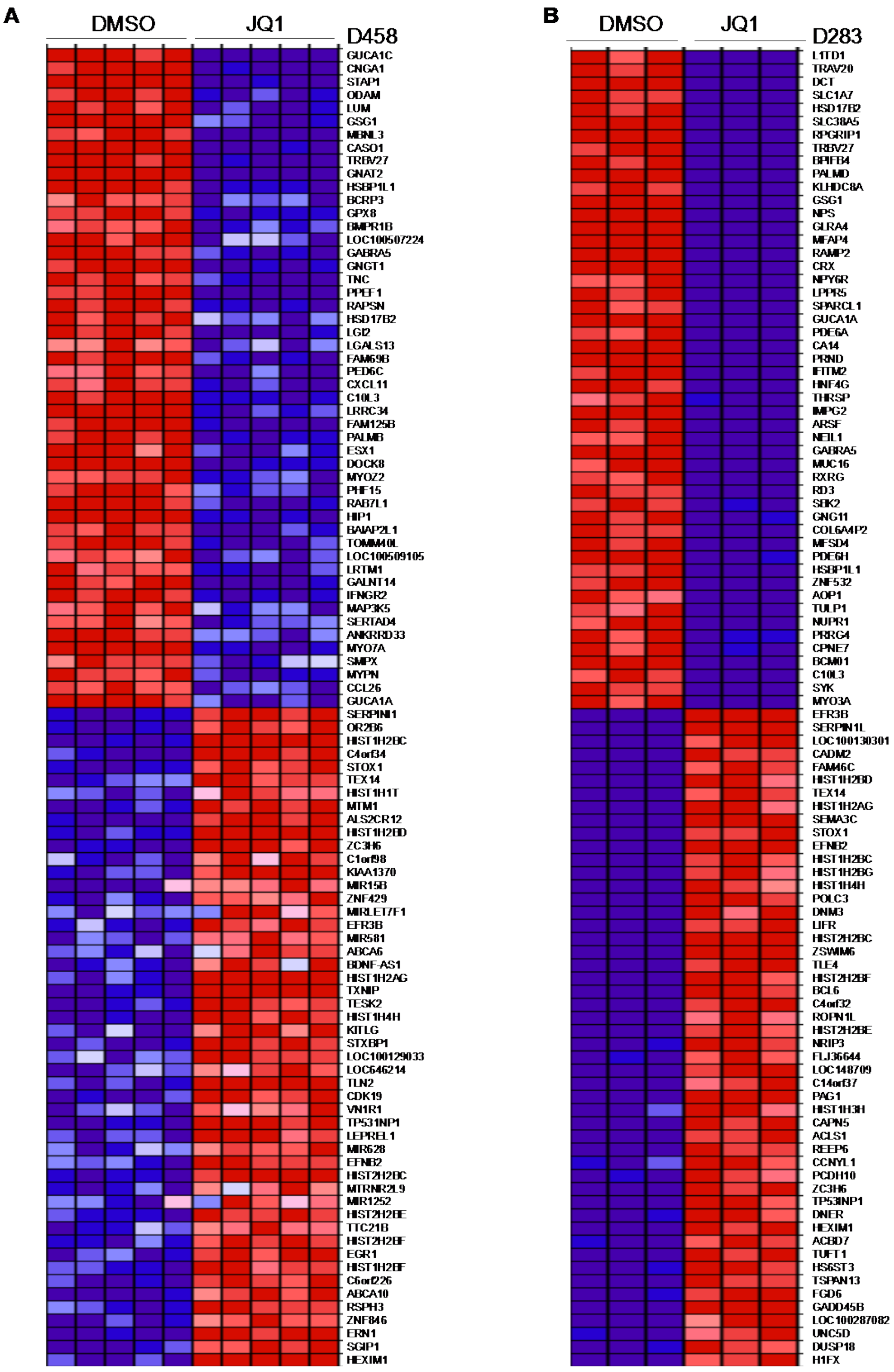

A
DMSO
JQ1
D458
GUCA1C
CNGA1
STAP1
ODAM
LUM
GSG1
MBNL3
CASQ1
TRBV27
GNAT2
HSBP1L1
BCRP3
GPX8
BMPR1B
LOC100507224
GABRA5
GNGT1
TNC
PPEF1
RAPSN
HSD17B2
LGI2
LGALS13
FAM69B
PDE6C
CXCL11
C10L3
LRRC34
FAM125B
PALMB
ESX1
DOCK8
MYOZ2
PHF15
RAB7L1
HIP1
BAIAP2L1
TOMM40L
LOC100509105
LRTM1
GALNT14
IFNGR2
MAP3K5
SERTAD4
ANKRRD33
MYO7A
SMPX
MYPN
CCL26
GUCA1A
SERPINI1
OR2B6
HIST1H2BC
C4orf34
STOX1
TEX14
HIST1H1T
MTM1
ALS2CR12
HIST1H2BD
ZC3H6
C1orf98
KIAA1370
MIR15B
ZNF429
MIRLET7F1
EFR3B
MIR581
ABCA6
BDNF-AS1
HIST1H2AG
TXNIP
TESK2
HIST1H4H
KITLG
STXBP1
LOC100129033
LOC646214
TLN2
CDK19
VN1R1
TP531NP1
LEPREL1
MIR628
EFNB2
HIST2H2BC
MTRNR2L9
MIR1252
HIST2H2BE
TTC21B
HIST2H2BF
EGR1
HIST1H2BF
C6orf226
ABCA10
RSPH3
ZNF846
ERN1
SGIP1
HEXIM1
B
DMSO
JQ1
D283
L1TD1
TRAV20
DCT
SLC1A7
HSD17B2
SLC38A5
RPGRIP1
TRBV27
BPIFB4
PALMD
KLHDC8A
GSG1
NPS
GLRA4
MFAP4
RAMP2
CRX
NPY6R
LPPR5
SPARCL1
GUCA1A
PDE6A
CA14
PRND
IFITM2
HNF4G
THRSP
IMPG2
ARSF
NEIL1
GABRA5
MUC16
RXRG
RD3
SIK2
GNG11
COL6A4P2
MFSD4
PDE6H
HSBP1L1
ZNF532
AOP1
TULP1
NUPR1
PRRG4
CPNE7
BCMO1
C10L3
SYK
MYO3A
EFR3B
SERPINI1L
LOC100130301
CADM2
FAM46C
HIST1H2BD
TEX14
HIST1H2AG
SEMA3C
STOX1
EFNB2
HIST1H2BC
HIST1H2BG
HIST1H4H
POLC3
DNM3
LIFR
HIST2H2BC
ZSWIM6
TLE4
HIST2H2BF
BCL6
C4orf32
ROPN1L
HIST2H2BE
NRIP3
FLJ36644
LOC148709
C14orf37
PAG1
HIST1H3H
CAPN5
ACLS1
REEP6
CCNYL1
PCDH10
ZC3H6
TP53INP1
DNER
HEXIM1
ACBD7
TUFT1
HS6ST3
TSPAN13
FGD6
GADD45B
LOC100287082
UNC5D
DUSP18
H1FX

Supplementary Figure 2

A. Gene expression heat map of top 50 differentially expressed genes between D425 cells treated with DMSO or JQ1 at a concentration of 1μM for 24 hours.

B. Gene expression heat map of top 50 differentially expressed genes between D341 cells treated with DMSO or JQ1 at a concentration of 1μM for 24 hours. Source data provided in Supplementary Table 1.

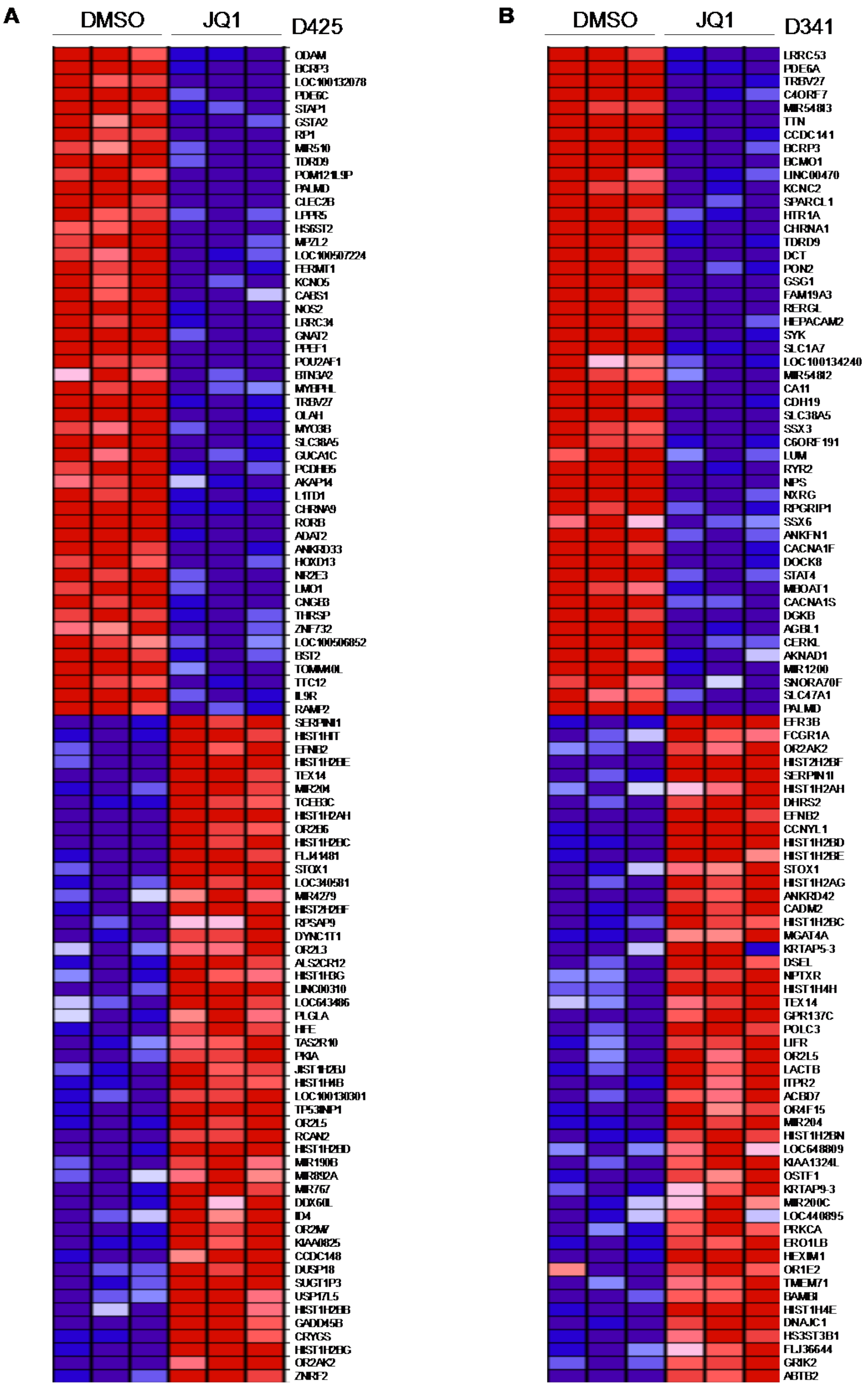
A
DMSO
JQ1
D425
ODAM
BCRP3
LOC100132078
PDE6C
STAP1
GSTA2
RP1
MIR510
TDRD9
POM121L9P
PALMD
CLEC2B
LPPR5
HS6ST2
MPZL2
LOC100507224
FERMT1
KCNO5
CABS1
NOS2
LRRC34
GNAT2
PPEF1
POU2AF1
BTN3A2
MYBPHL
TRBV27
OLAH
MYO3B
SLC38A5
GUCA1C
PCDHB5
AKAP14
L1TD1
CHRNA9
RORB
ADAT2
ANKRD33
HOXD13
NR2E3
LMO1
CNGB3
THRSP
ZNF732
LOC100506852
BST2
TOMM40L
TTC12
IL9R
RAMP2
SERPINI1
HIST1H1T
EFNB2
HIST1H2BE
TEX14
MIR204
TCEB3C
HIST1H2AH
OR2B6
HIST1H2BC
FLJ41481
STOX1
LOC340581
MIR4279
HIST2H2BF
RPSAP9
DYNC1I1
OR2L3
ALS2CR12
HIST1H3G
LINC00310
LOC643486
PLGLA
HFE
TAS2R10
PKIA
HIST1H2BJ
HIST1H4B
LOC100130301
TP53INP1
OR2L5
RCAN2
HIST1H2BD
MIR190B
MIR892A
MIR767
DDX60L
ID4
OR2M7
KIAA0825
CCDC148
DUSP18
SUGT1P3
USP17L5
HIST1H2BB
GADD45B
CRYGS
HIST1H2BG
OR2AK2
ZNRF2
B
DMSO
JQ1
D341
LRRC53
PDE6A
TRBV27
C4ORF7
MIR548I3
TTN
CCDC141
BCRP3
BCMO1
LINC00470
KCNC2
SPARCL1
HTR1A
CHRNA1
TDRD9
DCT
PON2
GSG1
FAM19A3
RERGL
HEPACAM2
SYK
SLC1A7
LOC100134240
MIR548I2
CA11
CDH19
SLC38A5
SSX3
C6ORF191
LUM
RYR2
NPS
NXRG
RPGRIP1
SSX6
ANKFN1
CACNA1F
DOCK8
STAT4
MBOAT1
CACNA1S
DGKB
AGBL1
CERKL
AKNAD1
MIR1200
SNORA70F
SLC47A1
PALMD
EFR3B
FCGR1A
OR2AK2
HIST2H2BF
SERPINI1
HIST1H2AH
DHRS2
EFNB2
CCNYL1
HIST1H2BD
HIST1H2BE
STOX1
HIST1H2AG
ANKRD42
CADM2
HIST1H2BC
MGAT4A
KRTAP5-3
DSEL
NPTXR
HIST1H4H
TEX14
GPR137C
POLC3
LIFR
OR2L5
LACTB
ITPR2
ACBD7
OR4F15
MIR204
HIST1H2BN
LOC648809
KIAA1324L
OSTF1
KRTAP9-3
MIR200C
LOC440895
PRKCA
ERO1LB
HEXIM1
OR1E2
TMEM71
BAMBI
HIST1H4E
DNAJC1
HS3ST3B1
FLJ36644
GRIK2
ABTB2

Supplementary Figure 3

A. Schematic depicting genome-scale CRISPR-Cas9 depletion screens to identify cell-essential genes.

B. Intersection of genes suppressed 24 hours following treatment with 1μM JQ1, relative to DMSO controls (blue), with those found to be cell essential (green) in D458 (top) and D283 (bottom) medulloblastoma cell lines by CRISPR-Cas9 screens. P-values indicate significance of overlap. P-values indicate significance of overlap as determined by Chi Square Test.

C. Schematic describing near genome-scale ORF screens to identify rescue genes in D283 and D458 cells treated with BET-bromodomain inhibitors (JQ1 and IBET151).

D. Correlation between log-fold-change of genes in near genome-scale ORF screens in D458 cells treated with JQ1 or IBET151. Significance of correlation determined by Pearson Correlation and p value is shown.

E. Correlation between log-fold change of genes in near genome-scale ORF screens in D283 cells treated with JQ1 or IBET151. . Significance of correlation determined by Pearson Correlation and p value is shown.

F. INTERPRO protein domains and GO pathways enriched in rescue genes that scored across ORF screens in both cell lines with either JQ1 or IBET151.

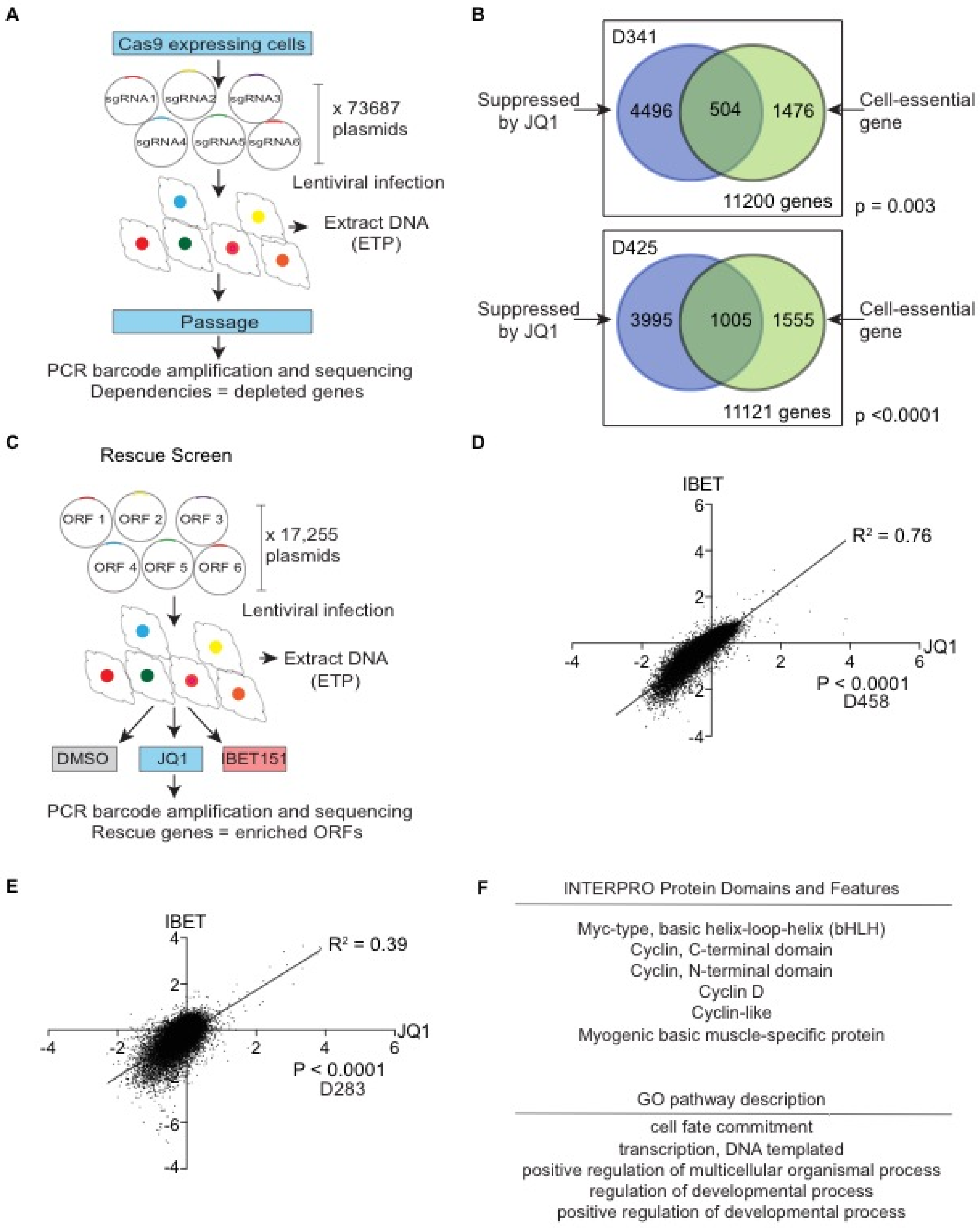
A
Cas9 expressing cells
sgRNA1
sgRNA2
sgRNA3
sgRNA4
sgRNA5
sgRNA6
x 73687 plasmids
Lentiviral infection
Extract DNA (ETP)
Passage
PCR barcode amplification and sequencing
Dependencies = depleted genes
B
D341
Suppressed by JQ1
4496
504
1476
Cell-essential gene
11200 genes
p = 0.003
D425
Suppressed by JQ1
3995
1005
1555
Cell-essential gene
11121 genes
p <0.0001
C
Rescue Screen
ORF 1
ORF 2
ORF 3
ORF 4
ORF 5
ORF 6
x 17,255 plasmids
Lentiviral infection
Extract DNA (ETP)
DMSO
JQ1
IBET151
PCR barcode amplification and sequencing
Rescue genes = enriched ORFs
D
IBET
JQ1
R² = 0.76
P < 0.0001
D458
E
IBET
JQ1
R² = 0.39
P < 0.0001
D283
F
INTERPRO Protein Domains and Features
Myc-type, basic helix-loop-helix (bHLH)
Cyclin, C-terminal domain
Cyclin, N-terminal domain
Cyclin D
Cyclin-like
Myogenic basic muscle-specific protein
GO pathway description
cell fate commitment
transcription, DNA templated
positive regulation of multicellular organismal process
regulation of developmental process
positive regulation of developmental process

Supplementary Figure 4

False discovery rates and dependency probabilities of genes subjected to CRISPR-cas9 pooled screens across D458 (A), D283 (B), D425 (C) and D341 (D) medulloblastoma cell lines. Essential genes were identified as those that had dependency scores of greater 0.35 and false discovery rates of less than 0.2 as indicated. Source data is provided in Supplementary Table 2.

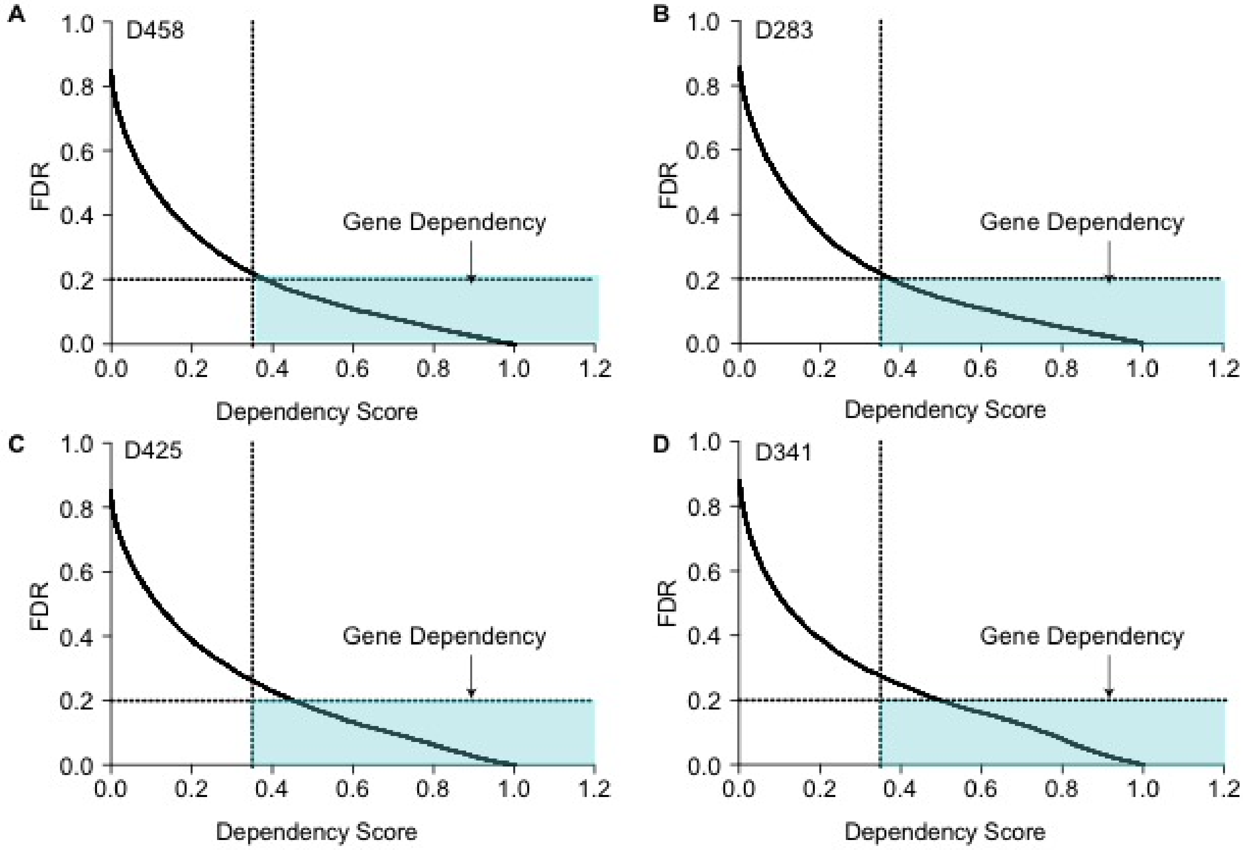
A
D458
B
D283
C
D425
D
D341
FDR
Gene Dependency
Dependency Score
0.0
0.2
0.4
0.6
0.8
1.0
1.2

Supplementary Figure 5

Top 50 gene-dependencies (without exclusion of pan-essential genes) as identified by CRISPR-cas9 screens across the D458 (A), D283 (B), D425 (C) and D341 (D) medulloblastoma cell lines. Source data is provided in Supplementary Table 2.

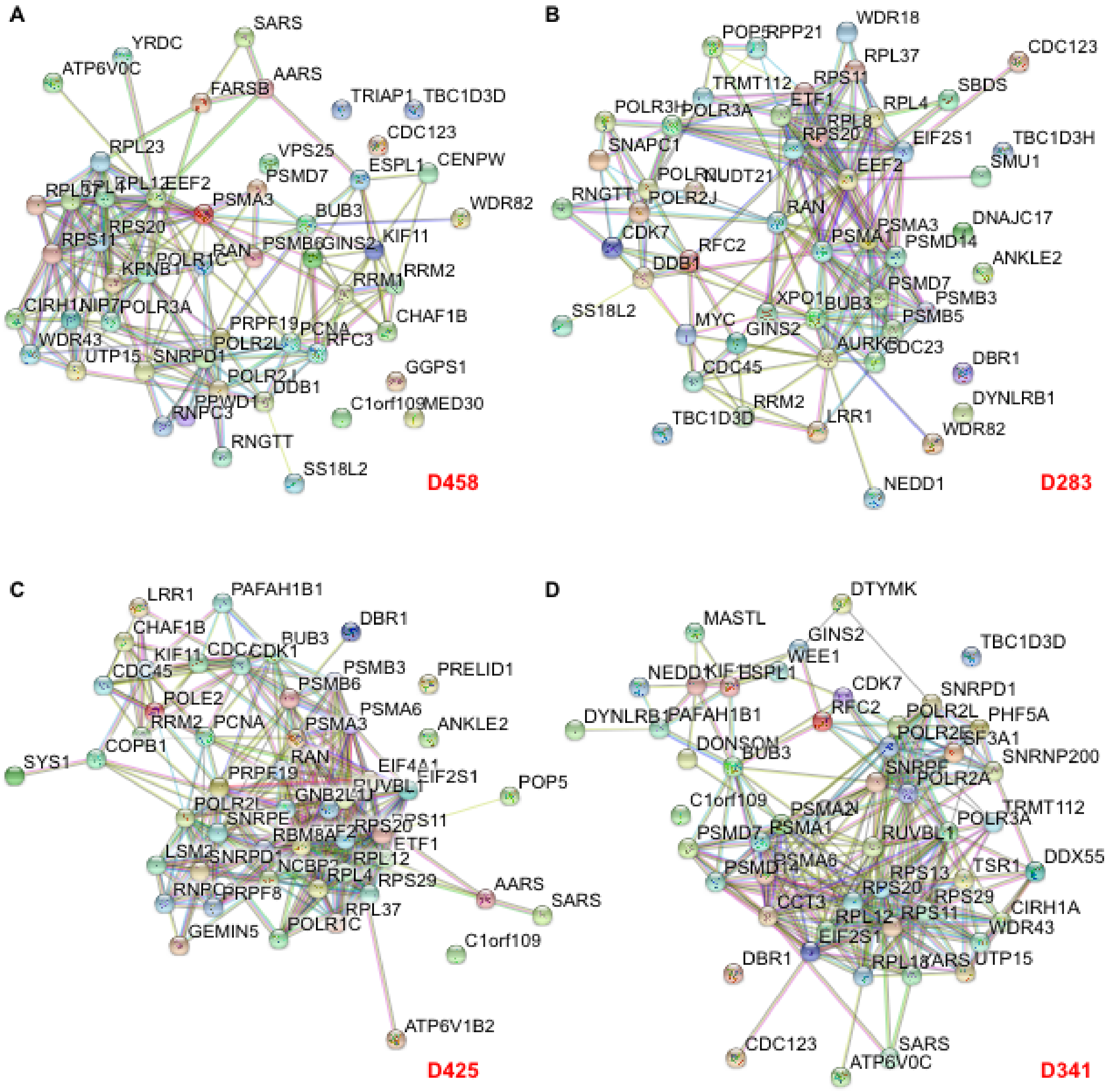
A
SARS
YRDC
ATP6V0C
AARS
FARSB
TRIAP1
TBC1D3D
CDC123
RPL23
VPS25
ESPL1
CENPW
PSMD7
EEF2
PSMA3
WDR82
BUB3
RPS20
RPS11
RAN
PSMB6
GINS2
KIF11
KPNB1
RRM1
RRM2
CIRH1
POLR3A
CHAF1B
PRPF19
PCNA
WDR43
UTP15
SNRPD1
RFC3
GGPS1
POLR2J
DDB1
MED30
RNPC3
RNGTT
SS18L2
D458
B
WDR18
CDC123
RPL37
RPS11
TRMT112
SBDS
POLR3H
POLR3A
ETF1
RPL4
RPL8
RPS20
EIF2S1
TBC1D3H
SNAPC1
SMU1
EEF2
NUDT21
RNGTT
POLR2J
RAN
CDK7
PSMA3
DNAJC17
PSMD14
RFC2
DDB1
ANKLE2
PSMD7
XPO1
BUB3
PSMB3
SS18L2
MYC
GINS2
PSMB5
CDC23
DBR1
CDC45
RRM2
DYNLRB1
TBC1D3D
LRR1
WDR82
NEDD1
D283
C
LRR1
PAFAH1B1
DBR1
CHAF1B
BUB3
KIF11
CDK1
PSMB3
CDC45
PRELID1
PSMB6
POLE2
PSMA6
RRM2
PCNA
PSMA3
ANKLE2
COPB1
SYS1
RAN
PRPF19
EIF2S1
POP5
SNRPE
ETF1
RPL12
RPL4
RPS29
AARS
SARS
PRPF8
RPL37
GEMIN5
POLR1C
C1orf109
ATP6V1B2
D425
D
DTYMK
MASTL
GINS2
TBC1D3D
WEE1
ESPL1
CDK7
SNRPD1
DYNLRB1
PAFAH1B1
RFC2
POLR2L
PHF5A
SF3A1
DONSON
BUB3
SNRNP200
POLR2A
C1orf109
TRMT112
PSMA1
RUVBL1
POLR3A
PSMD7
PSMA6
TSR1
DDX55
RPS13
RPS20
CCT3
RPS29
CIRH1A
RPL12
RPS11
WDR43
EIF2S1
DBR1
UTP15
CDC123
SARS
ATP6V0C
D341

Supplementary Figure 6

A. Representative immunoblots of expression of indicated V5-tagged ORFs in D458 and D283 cell lines used in proliferation assays. Source data provided as a Source Data File.

B. Representative immunoblots of expression of indicated V5-tagged ORFs in D458 and D283 cell lines used in annexin V/propidium iodide apoptosis assays. Source data provided as a Source Data File.

C. Representative immunoblots of expression of indicated V5-tagged ORFs in D341, CHLA01 and MB002 cell lines used in proliferation assays. Source data provided as a Source Data File.

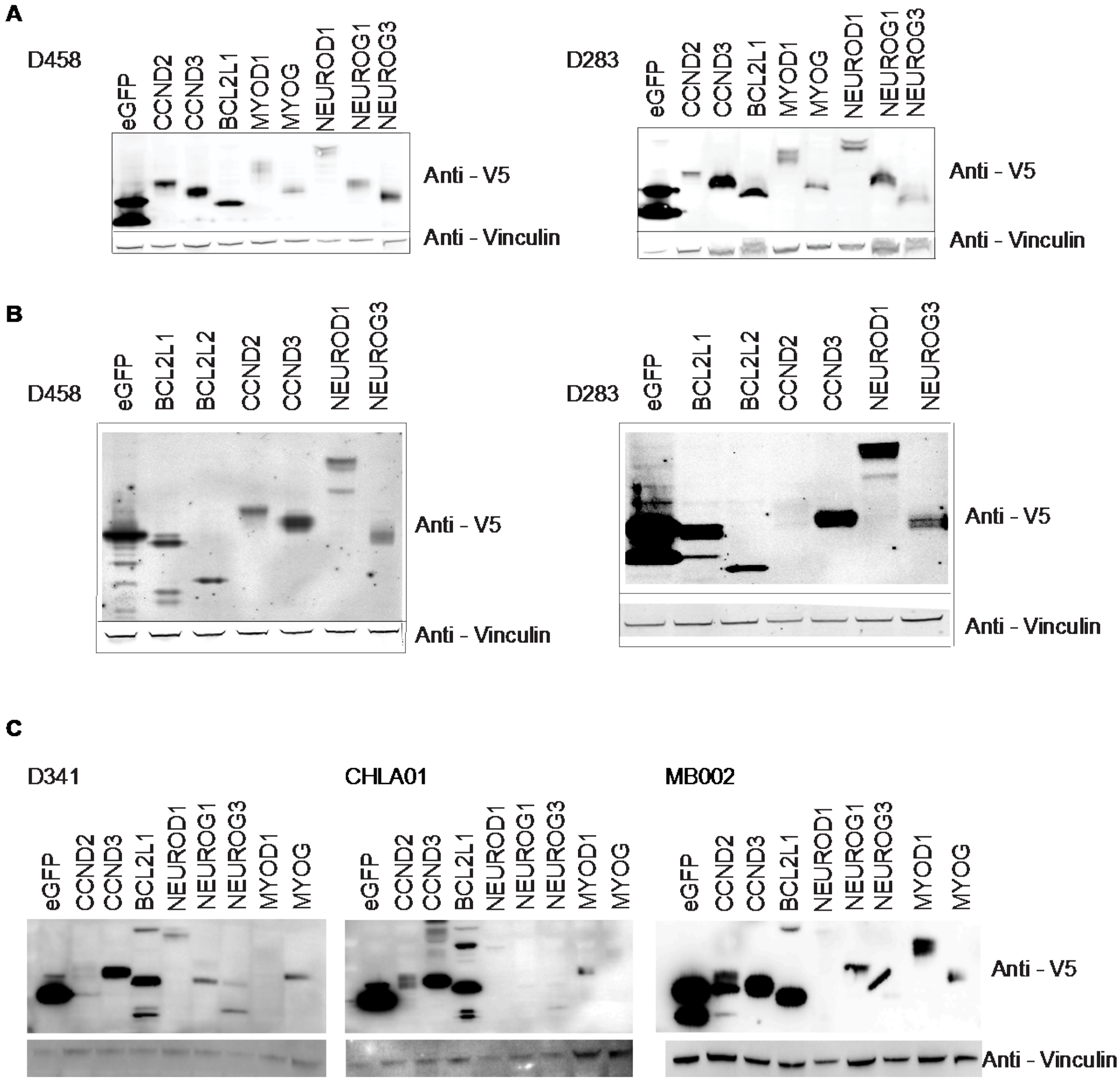
A
D458
eGFP
CCND2
CCND3
BCL2L1
MYOD1
MYOG
NEUROD1
NEUROG1
NEUROG3
Anti - V5
Anti - Vinculin
D283
eGFP
CCND2
CCND3
BCL2L1
MYOD1
MYOG
NEUROD1
NEUROG1
NEUROG3
Anti - V5
Anti - Vinculin
B
D458
eGFP
BCL2L1
BCL2L2
CCND2
CCND3
NEUROD1
NEUROG3
Anti - V5
Anti - Vinculin
D283
eGFP
BCL2L1
BCL2L2
CCND2
CCND3
NEUROD1
NEUROG3
Anti - V5
Anti - Vinculin
C
D341
eGFP
CCND2
CCND3
BCL2L1
NEUROD1
NEUROG1
NEUROG3
MYOD1
MYOG
CHLA01
eGFP
CCND2
CCND3
BCL2L1
NEUROD1
NEUROG1
NEUROG3
MYOD1
MYOG
MB002
eGFP
CCND2
CCND3
BCL2L1
NEUROD1
NEUROG1
NEUROG3
MYOD1
MYOG
Anti - V5
Anti - Vinculin

Supplementary Figure 7

A-C. Low throughput rescue assays in MB002, D341 or CHLA01 cells expressing eGFP, CCND2, CCND3, BCL2L1, NEUROD1, NEUROG3, MYOD1 or MYOG that were treated with JQ1 1μM or DMSO control. Vertical axis indicates cumulative doubling of JQ1 treated cells relative to DMSO control. Asterisks denote statistically significant differences compared to eGFP (* $p<0.05$, ** $p<0.01$, *** $p<0.001$) as determined by two-tailed unpaired T-tests. Source data provided as a Source Data File.

D-H. Cumulative doublings of D458, D283, MB002, D341 and CHLA01 expressing indicated ORFs passaged in DMSO for seven days. Data from three independent experiments in each line are shown; error bars depict mean ± SEM. Asterisks denote statistically significant differences compared to eGFP (* $p<0.05$, ** $p<0.01$, *** $p<0.001$) as determined by two-tailed unpaired T-tests. Source data provided as a Source Data File.

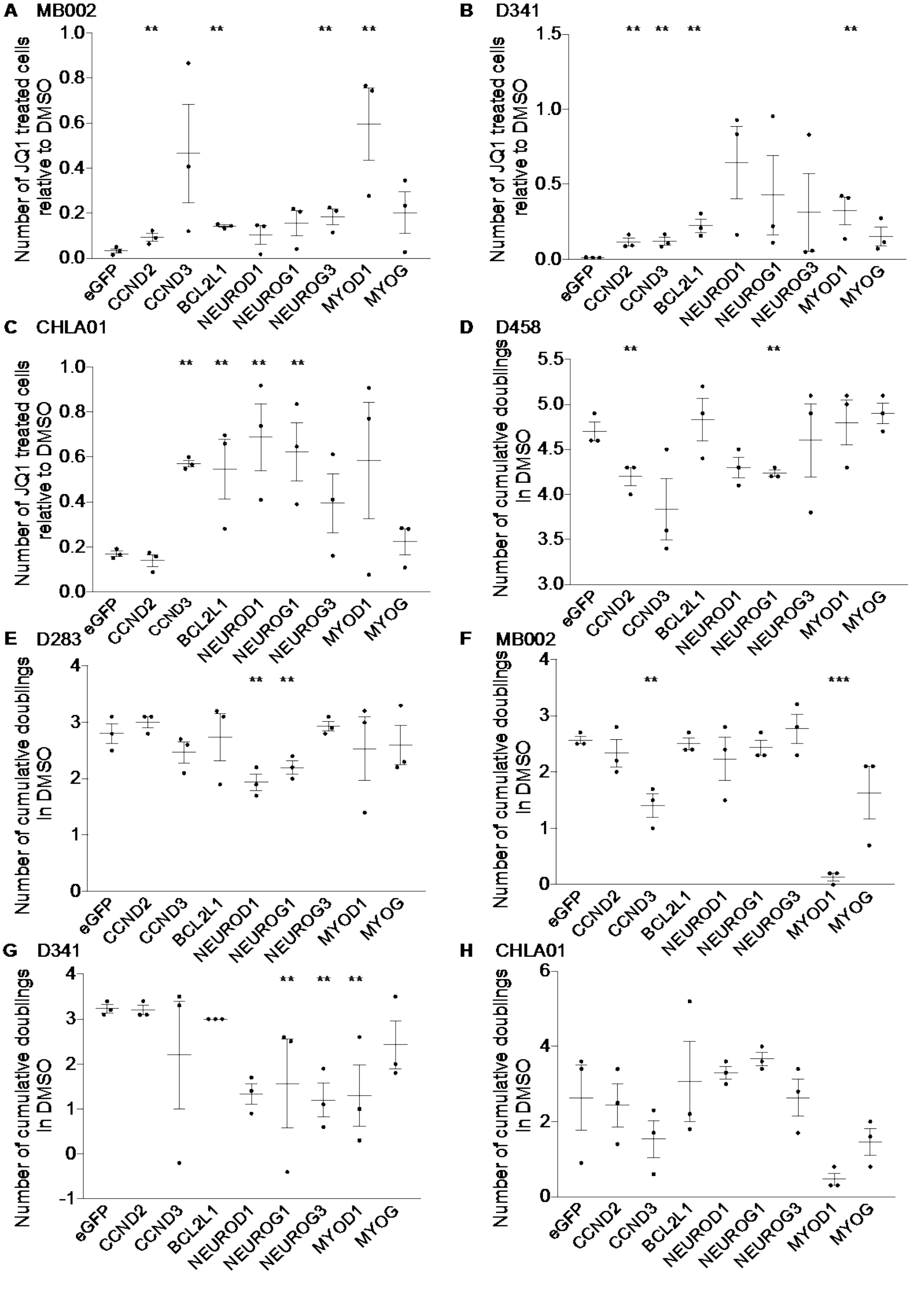

Supplementary Figure 8

A. Proliferation of sensitive and drug-tolerant D425 and D458 medulloblastoma cells in JQ1 or IBET151 (or vehicle control). Values represent mean independent experiments ± SEM in each cell line. Asterisks denote statistically significant differences (* p<0.05, ** p<0.01, *** p<0.001) as determined by two-tailed unpaired T-tests. Source data provided as a Source Data File.

B. Percentage of apoptotic (Annexin V positive) medulloblastoma cells treated with JQ1 or vehicle controls. Values represent mean of six independent experiments (across D425 and D458 sensitive and drug-tolerant cells) ± SEM. Asterisks denote statistically significant differences (* p<0.05, ** p<0.01, *** p<0.001) as determined by two-tailed unpaired T-tests. Source data provided as a Source Data File.

C. Percentage of necrotic cells (Propidium iodide positive, Annexin V negative) medulloblastoma cells treated with JQ1 or vehicle controls. Values represent mean of six independent experiments (across D425 and D458 sensitive and drug-tolerant cells) ± SEM. Asterisks denote statistically significant differences (* p<0.05, ** p<0.01, *** p<0.001) as determined by two-tailed unpaired T-test. Source data provided as a Source Data File.

D. Venn diagram depicting overlap of genes suppressed by JQ1 in D458 drug naive medulloblastoma cells and genes upregulated in D458 drug tolerant cells. P value was determined by two-tailed Chi square test.

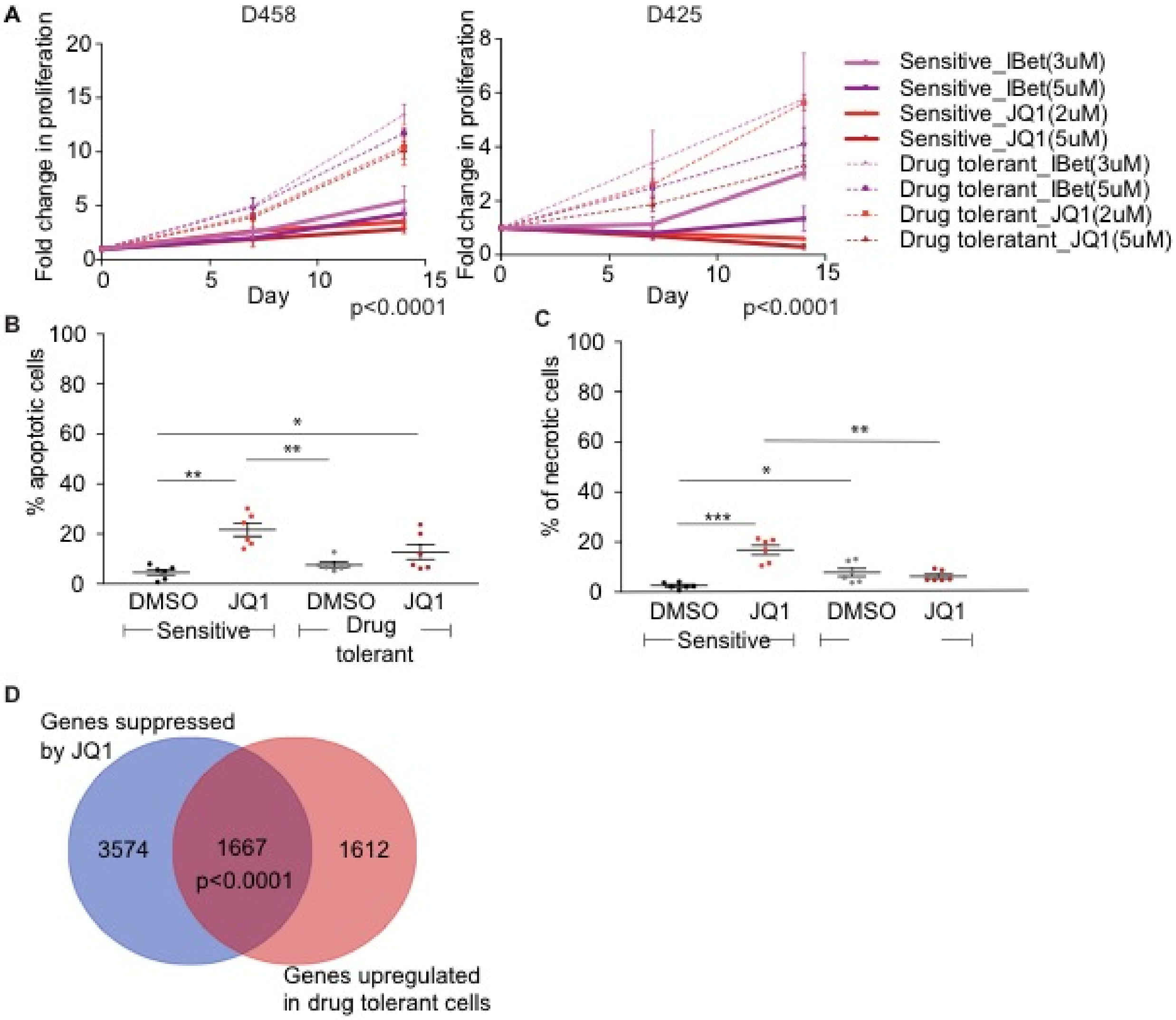
A
D458
Fold change in proliferation
20
15
10
5
0
0
5
10
15
Day
p<0.0001
D425
8
6
4
2
0
Sensitive_IBet(3uM)
Sensitive_IBet(5uM)
Sensitive_JQ1(2uM)
Sensitive_JQ1(5uM)
Drug tolerant_IBet(3uM)
Drug tolerant_IBet(5uM)
Drug tolerant_JQ1(2uM)
Drug toleratant_JQ1(5uM)
B
% apoptotic cells
100
80
60
40
20
DMSO
JQ1
Sensitive
Drug tolerant
*
**
C
% of necrotic cells
***
D
Genes suppressed by JQ1
3574
1667
p<0.0001
1612
Genes upregulated in drug tolerant cells

Supplementary Figure 9

A-B. GSEA depicting upregulation of MYC activation pathways (A) and E2F targets (B) in drug-tolerant D458 cells (n=5) relative to sensitive cells (n=5).

C-E. Fold change in densitometry measurements of D458 sensitive and drug-tolerant cells treated with DMSO or 1uM JQ1 for 24 hours, probing for CCND2 (C), BCL2L1 (D) or HLX (E), normalized to vinculin loading control. Values represent mean of three independent immunoblots ± SEM. Asterisks denote statistically significant differences (* $p<0.05$, ** $p<0.01$, *** $p<0.001$) as determined by two-tailed unpaired T-test. Source data provided as a Source Data File.

F-G. Fold change in densitometry measurements of D458 sensitive and drug-tolerant cells treated with DMSO or 1uM JQ1 for 24 hours, probing for NEUROD1 (F) and NEUROG1 (G), normalized to vinculin loading control. Values represent mean of three independent immunoblots ± SEM. Asterisks denote statistically significant differences (* $p<0.05$, ** $p<0.01$, *** $p<0.001$) as determined by two-tailed unpaired T-test. Source data provided as a Source Data File.

H. Fold change in densitometry measurements of mouse neural stem cells transduced to express one of eGFP, MYOD1, MYOG, NEUROD1 and NEUROG1, probing for TUJ1, normalized to vinculin loading control. Values represent mean of three independent immunoblots ± SEM. Asterisks denote statistically significant differences (* $p<0.05$, ** $p<0.01$, *** $p<0.001$) as determined by two-tailed unpaired T-test. Source data provided as a Source Data File.

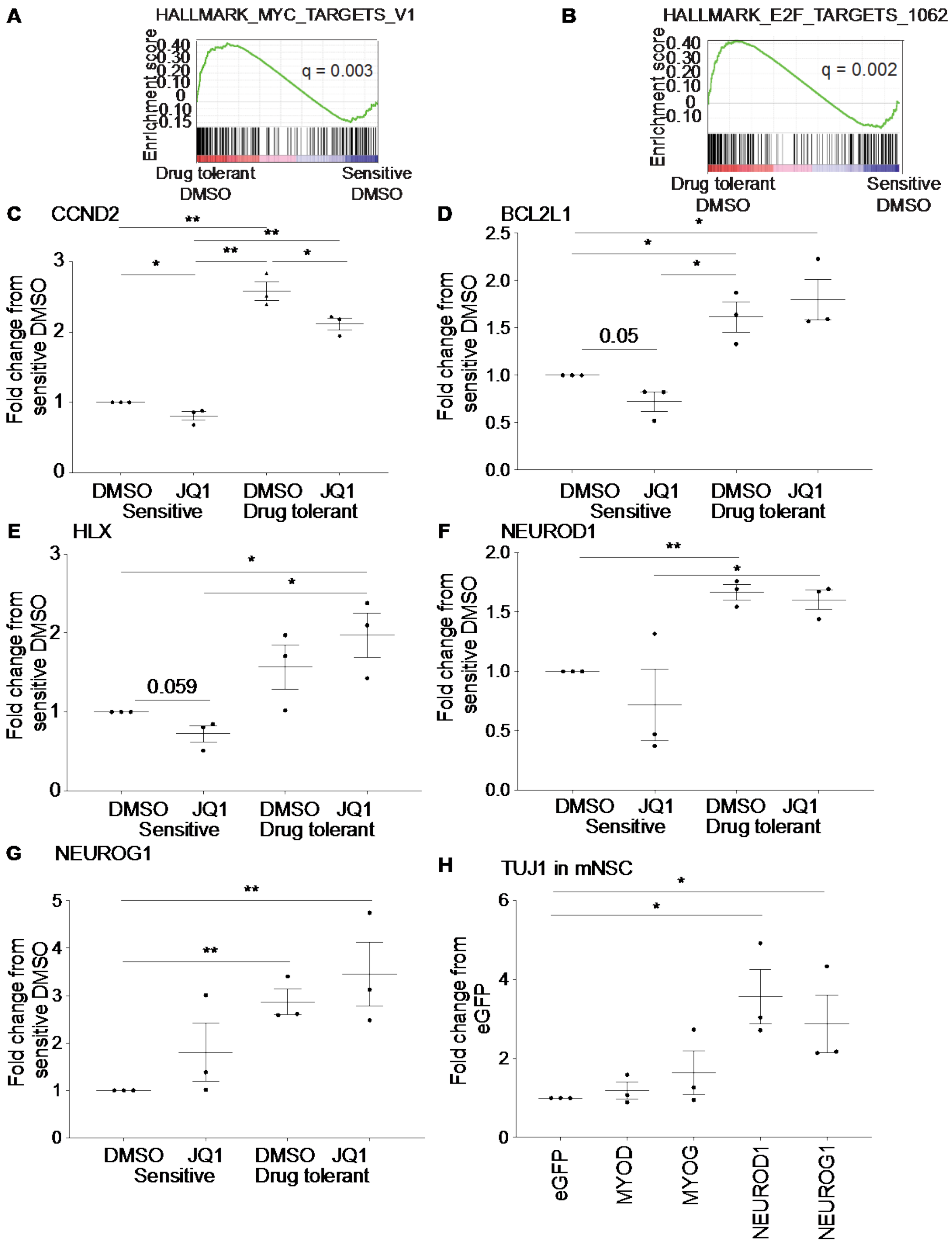

A
HALLMARK_MYC_TARGETS_V1
Enrichment score
q = 0.003
Drug tolerant DMSO
Sensitive DMSO
B
HALLMARK_E2F_TARGETS_1062
Enrichment score
q = 0.002
Drug tolerant DMSO
Sensitive DMSO
C
CCND2
Fold change from sensitive DMSO
DMSO JQ1 Sensitive
DMSO JQ1 Drug tolerant
D
BCL2L1
0.05
Fold change from sensitive DMSO
DMSO JQ1 Sensitive
DMSO JQ1 Drug tolerant
E
HLX
0.059
Fold change from sensitive DMSO
DMSO JQ1 Sensitive
DMSO JQ1 Drug tolerant
F
NEUROD1
Fold change from sensitive DMSO
DMSO JQ1 Sensitive
DMSO JQ1 Drug tolerant
G
NEUROG1
Fold change from sensitive DMSO
DMSO JQ1 Sensitive
DMSO JQ1 Drug tolerant
H
TUJ1 in mNSC
Fold change from eGFP
eGFP
MYOD
MYOG
NEUROD1
NEUROG1

Supplementary Figure 10

A-D Fold change in densitometry measurements of D458 sensitive and drug-tolerant cells treated with DMSO or 1uM JQ1 for 24 hours, probing for TUJ1 (A), NF68 (B), MSI1 (C) and SOX2 (D), normalized to vinculin loading control. Values represent mean of three independent immunoblots ± SEM. Asterisks denote statistically significant differences (* $p<0.05$, ** $p<0.01$, *** $p<0.001$) as determined by two-tailed unpaired T-test. Source data provided as a Source Data File.

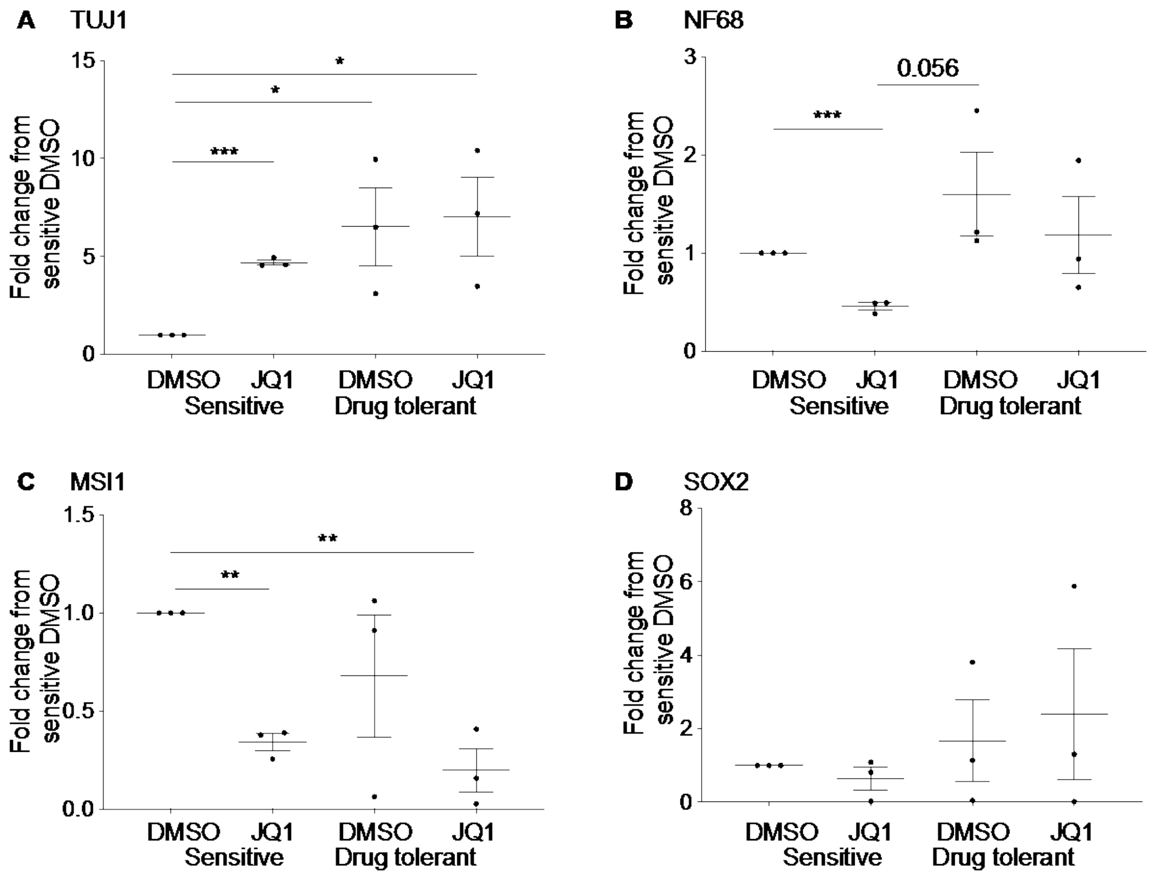

A
TUJ1
Fold change from sensitive DMSO
15
10
5
0
***
*
*
DMSO
JQ1
DMSO
JQ1
Sensitive
Drug tolerant
B
NF68
3
2
1
0
***
0.056
C
MSI1
1.5
1.0
0.5
0.0
**
**
D
SOX2
8
6
4
2
0

Supplementary Figure 11

A. Representative immunoblots probing for MYC in D425 and D458 sensitive and drug-tolerant cells treated with JQ1 or vehicle control for 24 hours are shown. Source data provided as Source Data File.

B. Expression of rescue genes identified in D458 ORF screen in D458 sensitive and drug-tolerant cells passaged in DMSO or JQ1 (1μM) for 24 hours. Red indicates high expression and blue indicates low expression. Genes shown in red represent those identified to be suppressed by JQ1 (q value <0.1) and re-expressed in drug-tolerant cells (q value <0.1) as identified by gene-expression profiling.

C-D. Representative immunoblots of D458 sensitive and drug tolerant cells transduced with lentiviral vectors expressing short hairpins targeting *GFP* (negative control), *SF3B1* (positive control), *BCL2L1, CCND2 or NEUROD1*. Source data provided in Source Data File.

E. ChIP-seq binding scores (Z-transformed) of H3K4me3 and H3K27ac marks to the genes that did not score in ORF screens in sensitive cells treated with DMSO or 1μM JQ1 and drug-tolerant cells passaged in 1μM JQ1. Data from four independent experiments in each line are shown (3 replicates for H3K27ac binding drug tolerant cells); error bars depict mean ± SEM. Asterisks denote significant differences (* $p<0.05$, ** $p<0.01$, ** $p<0.0001$). Source data are included as a Source Data File.

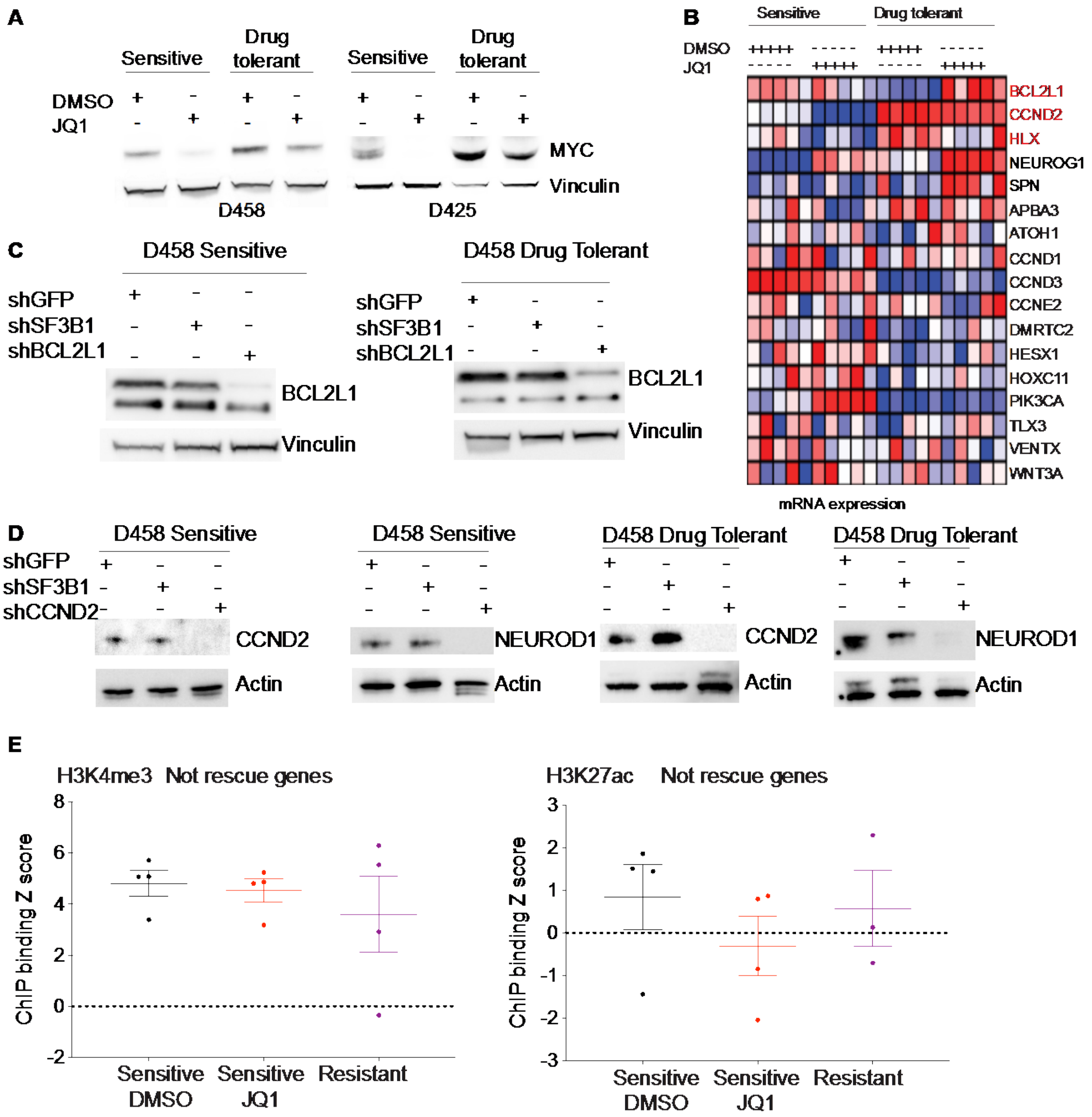

A
Sensitive
Drug tolerant
Sensitive
Drug tolerant
DMSO
JQ1
MYC
Vinculin
D458
D425
B
Sensitive
Drug tolerant
DMSO
JQ1
BCL2L1
CCND2
HLX
NEUROG1
SPN
APBA3
ATOH1
CCND1
CCND3
CCNE2
DMRTC2
HESX1
HOXC11
PIK3CA
TLX3
VENTX
WNT3A
mRNA expression
C
D458 Sensitive
D458 Drug Tolerant
shGFP
shSF3B1
shBCL2L1
BCL2L1
Vinculin
D
D458 Sensitive
D458 Drug Tolerant
shGFP
shSF3B1
shCCND2
CCND2
NEUROD1
Actin
E
H3K4me3 Not rescue genes
H3K27ac Not rescue genes
ChIP binding Z score
Sensitive DMSO
Sensitive JQ1
Resistant

Supplementary Figure 12

A. Top blot: Lysates derived from mouse neural stem cells and probed for TUJ1 (or vinculin control) 7 days following overexpression of indicated bHLH transcription factors (or eGFP controls). Bottom blot: Lysates obtained from the same cells at 72 hours were also probed with V5 to confirm overexpression of individual ORFs.

B-F. Fold change in cell-number (relative to baseline) of CHLA01, D458, MB002, D341, and D283 cells treated with JQ1, LEE011 or combination of both compounds, at the concentrations indicated. Data from three independent experiments in each line are shown; error bars depict mean ± SEM. Asterisks denote significant differences from eGFP controls (* $p<0.05$, ** $p<0.01$, ** $p<0.0001$). Red asterisks denote concentrations that meet the Bliss criteria for synergy.

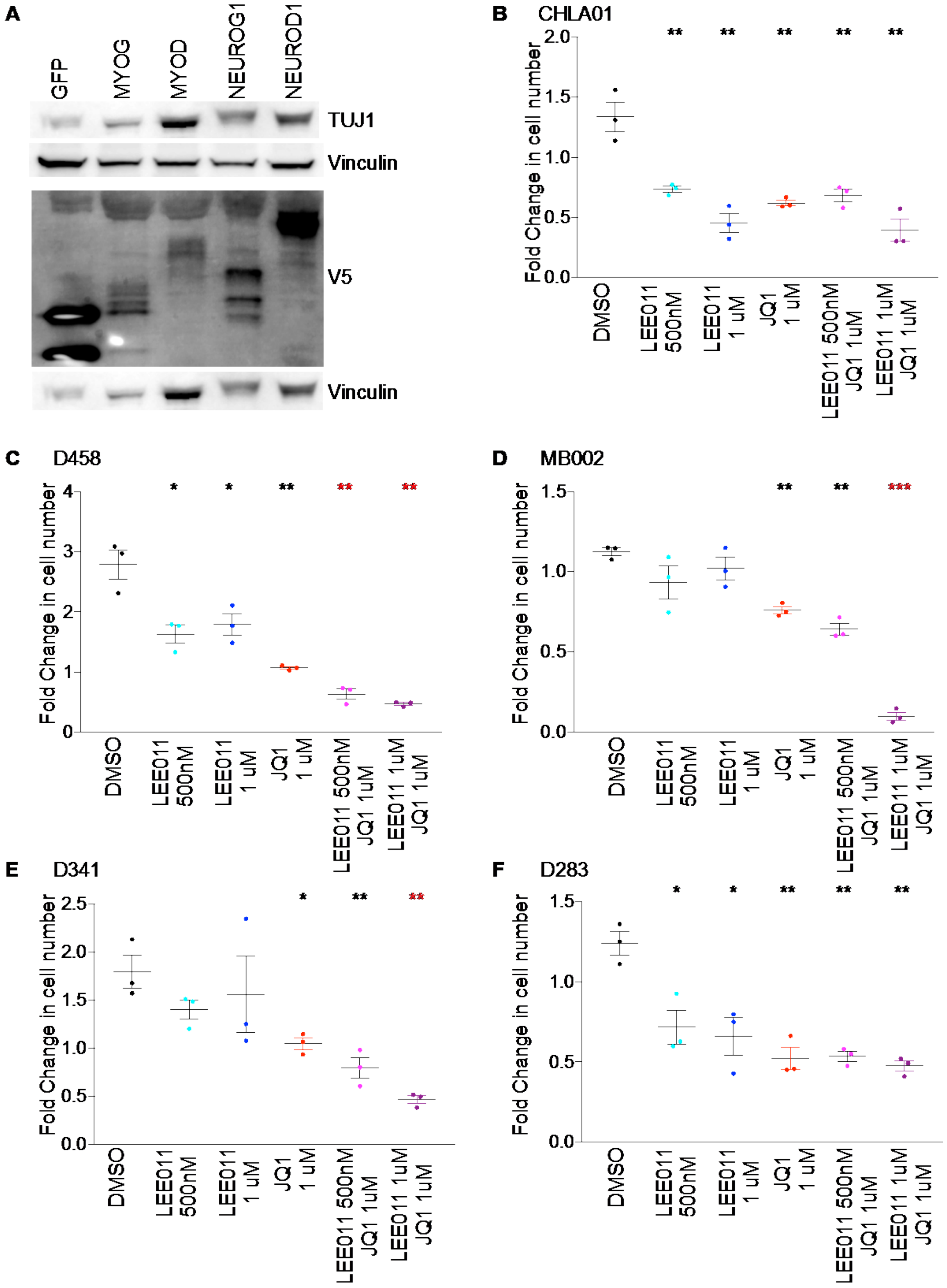
A
GFP
MYOG
MYOD
NEUROG1
NEUROD1
TUJ1
Vinculin
V5
Vinculin
B
CHLA01
** ** ** ** **
2.0
1.5
1.0
0.5
0.0
Fold Change in cell number
DMSO
LEE011 500nM
LEE011 1 uM
JQ1 1 uM
LEE011 500nM JQ1 1uM
LEE011 1uM JQ1 1uM
C
D458
* * ** ** **
4
3
2
1
0
Fold Change in cell number
DMSO
LEE011 500nM
LEE011 1 uM
JQ1 1 uM
LEE011 500nM JQ1 1uM
LEE011 1uM JQ1 1uM
D
MB002
** ** ***
1.5
1.0
0.5
0.0
Fold Change in cell number
DMSO
LEE011 500nM
LEE011 1 uM
JQ1 1 uM
LEE011 500nM JQ1 1uM
LEE011 1uM JQ1 1uM
E
D341
* ** **
2.5
2.0
1.5
1.0
0.5
0.0
Fold Change in cell number
DMSO
LEE011 500nM
LEE011 1 uM
JQ1 1 uM
LEE011 500nM JQ1 1uM
LEE011 1uM JQ1 1uM
F
D283
* * ** ** **
1.5
1.0
0.5
0.0
Fold Change in cell number
DMSO
LEE011 500nM
LEE011 1 uM
JQ1 1 uM
LEE011 500nM JQ1 1uM
LEE011 1uM JQ1 1uM

Supplementary Figure 13

Response rates of HD-MB003 (A), D283 (B), D458 (C) and MB002 (D) cell lines to single agent JQ1 and LEE011 (top panels), and in combination (heat maps). Effect size is plotted on the Y axis for single agents. A score of 0 indicates no effect on viability, 100% denotes a cytostatic effect and 200% denotes a cytotoxic effect. Heatmaps indicate levels of synergy (red) or antagonism (blue) of the combination of JQ1 and LEE011 at the concentrations shown, as determined by Loewe’s synergy model.

B

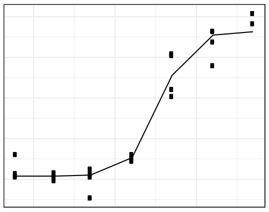

3

Supplementary Figure 14

A. Response rates of CHLA01 to single agent JQ1 and LEE011 (top panels), and in combination (heat maps). Effect size is plotted on the Y axis for single agents. A score of 0 indicates no effect on viability, 100% denotes a cytostatic effect and 200% denotes a cytotoxic effect. Heatmaps indicate levels of synergy (red) or antagonism (blue) of the combination of JQ1 and LEE011 at the concentrations shown, as determined by Loewe's synergy model.

B. Bioluminescence values of D458 intracranial xenografts in mice treated with vehicle, JQ1 (50mg/kg/day), LEE011 (75mg/kg/day) or the combination of both compounds (JQ1 50mg/kg/day and LEE011 75mg/kg/day). Data represent mean tumor volume normalized to day 1, ± SEM. * denotes $p<0.05$.

C. Expression levels of TUJ1 (TUBB3) and JQ1 sensitivity of 783 cell lines from the CTRP database. AUC denotes area under the curve. Asterisks denote statistically significant differences (* $p<0.05$, ** $p<0.01$, *** $p<0.001$).

D. Expression levels of TUJ1 (TUBB3) and JQ1 sensitivity of 7 prostate cell lines from the CTRP database. AUC denotes area under the curve. Asterisks denote statistically significant differences (* $p<0.05$, ** $p<0.01$, *** $p<0.001$).

E. Expression levels of TUJ1 (TUBB3) and JQ1 sensitivity of 11 thyroid cell lines from the CTRP database. AUC denotes area under the curve. Asterisks denote statistically significant differences (* $p<0.05$, ** $p<0.01$, *** $p<0.001$).

F. Percentage of shared DNA barcodes across replicates of barcoded D283 cells treated with JQ1(2μM). Within each replicate, the number of other replicates with whom each barcode is shared is depicted.

G. Heatmap showing Pearson correlations between replicates of barcoded D458 cells treated with JQ1 or DMSO. ETP denotes early time point control.

H. Heatmap showing Pearson correlations between replicates of barcoded D283 cells treated with JQ1 or DMSO. ETP denotes early time point control.

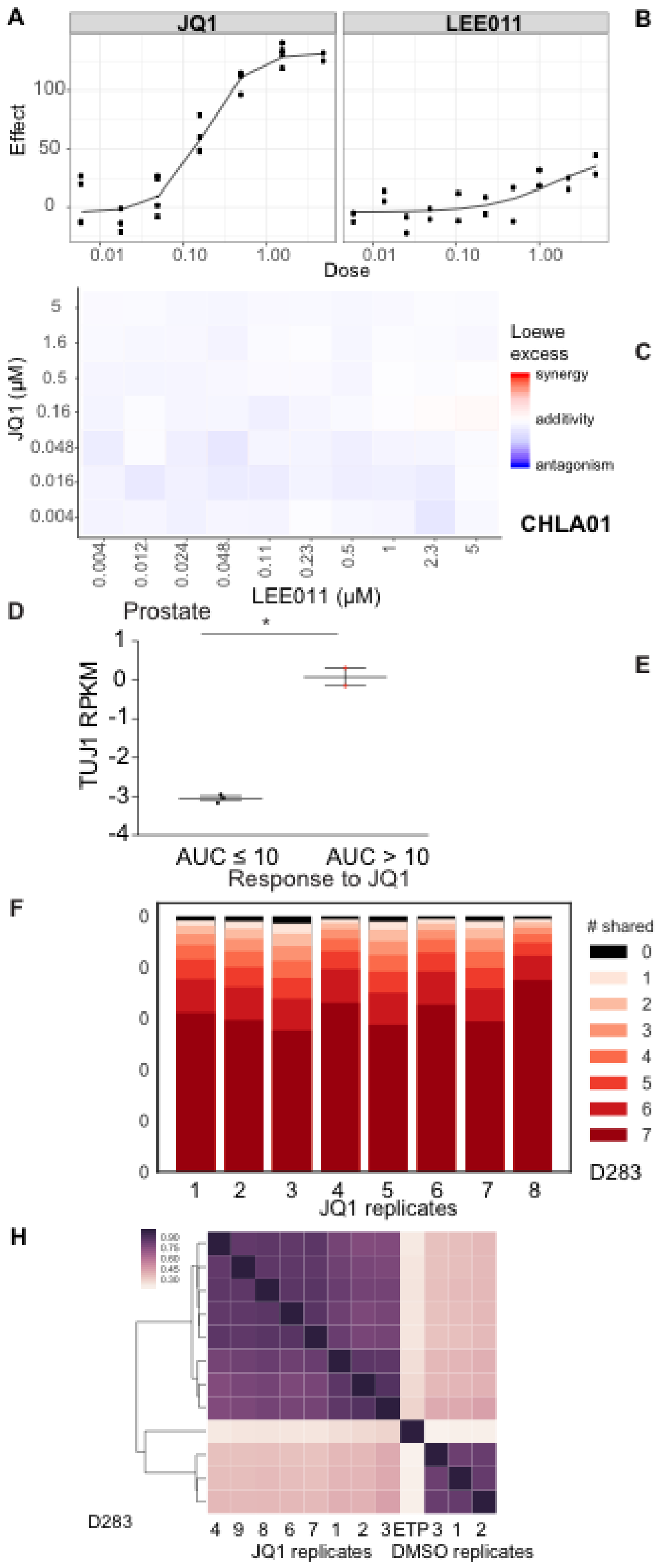

A
JQ1
LEE011
Effect
Dose
JQ1 (µM)
LEE011 (µM)
Loewe excess
synergy
additivity
antagonism
CHLA01
B
D458 Intracranial
Bioluminescence
Days
C
All cancer cell lines
TUJ1 RPKM
AUC ≤ 10
AUC > 10
D
Prostate
TUJ1 RPKM
AUC ≤ 10
AUC > 10
Response to JQ1
E
Thyroid
TUJ1 RPKM
AUC ≤ 10
AUC > 10
Response to JQ1
F
shared
D283
JQ1 replicates
G
D458
JQ1
DMSO
H
D283
JQ1 replicates
DMSO replicates
