# Supplementary material for: Neuronal differentiation and cell-cycle programs mediate response to BET-bromodomain inhibition in MYC-driven medulloblastoma
Source: Nat Commun. 2019 Jun 3;10:2400. doi: 10.1038/s41467-019-10307-9 (PMC6546744; doi:10.1038/s41467-019-10307-9)
Supplement: Supplementary file 2 — Description of Additional Supplementary Files [file 41467_2019_10307_MOESM2_ESM.docx]

**Figure Legends for Supplementary Data Files:**

Supplementary Data 1

Comparative marker selection analysis of D283, D458, D341 and D425 cells treated with JQ1 for 24 hours. Genes that are upregulated in DMSO controls (and suppressed in JQ1 treated cells) are shown.

Supplementary Data 2

Genome-scale dependency probabilities and false discovery rates of D283, D458, D341 and D425 cells as determined by CRISPR-Cas9 screening.

Supplementary Data 3

Pathways enriched in genetic dependencies that are exploited by BETi. Gene-set enrichment analysis (using the C2CP pathways) was applied to identify pathways that are suppressed by BETi in cell-essential genes. Individual genes within each pathway are also included for reference.

Supplementary Data 4

Log-fold changes of ORFs in D458 and D283 genome-scale ORF screens in the presence of JQ1 or IBET151. Rescue hits that scored with relaxed thresholds (log fold change >1.1 and q value <0.25) are shown for both cell lines in the presence of either JQ1 or IBET151.

Supplementary Data 5

A. Comparative marker selection identifies differentially expressed genes in D458 sensitive cells (n=5) in 1μM of JQ1 for 24 hours relative to drug-tolerant D458 cells (n=5), also passaged in 1μM of JQ1.

B. Expression of JQ1 consensus genes in D458 sensitive and D458 drug-tolerant medulloblastoma cells.

Supplementary Data 6

Number of insertion/deletions (indels) and single nucleotide variants (SNPs) in D458 drug-tolerant cells and vehicle controls.

Supplementary Data 7

Mutation annotation files for indels and SNPs detected in D458 and D425 drug-tolerant cells and vehicle controls.

Supplementary Data 8

A. Differentially altered chromatin marks in drug-tolerant D458 cells as determined by global chromatin profiling.

B. Relative abundance and differential levels of all chromatin marks included in Global Chromatin Profiling assay as determined by comparative marker selection.

Supplementary Data 9

Proportion of medulloblastoma cells that express lineage and differentiation markers as determined by CycIF across two tissue microarrays.

Supplementary Data 10

A. Antibodies used for immunoblotting, flow cytometry and CycIF.

B. Primers used to generate DNA-barcoding library.
